# Identification of Chromatin Regulatory Factors Related to Immunity and Treatment of Alzheimer’s Disease

**DOI:** 10.1007/s12031-023-02107-0

**Published:** 2023-02-24

**Authors:** Fengzhen Xiong, Chenglong Li, Qingbo Wang, Xin Geng, Zhengbo Yuan, Zefu Li

**Affiliations:** Department of Neurosurgery, Binzhou Medical University Hospital, Binzhou, 256603 Shandong China

**Keywords:** GEO, Chromatin regulators, Alzheimer’s disease, Bioinformatics analysis, Immunotherapy

## Abstract

Alzheimer’s disease is one of the common neurodegenerative diseases in the elderly, which mainly manifests as progressively severe cognitive impairment, which seriously affects the quality of life of patients. Chromatin regulators have been shown to be associated with a variety of biological processes, and we mainly explore the relationship between chromatin regulators and Alzheimer’s disease. Eight hundred seventy chromatin regulators were collected from previous studies, and data related to Alzheimer’s disease patients were downloaded from the GEO database. Finally, we screened chromatin regulators related to Alzheimer's disease immunity, established prediction models, and screened related drugs and miRNAs. We screened 160 differentially expressed CRs, constructed an interaction network, obtained 10 hub genes, successfully constructed a prediction model based on immune-related 5 CRs, and obtained 520 related drugs and 3 related miRNA, which provided an idea for the treatment of Alzheimer's disease. Our study identified 5 chromatin regulators related to Alzheimer’s disease, which are expected to be new targets for Alzheimer’s disease immunotherapy.

## Introduction

Alzheimer’s disease (AD) is a persistent neurological disorder, which often occurs in the elderly or pre-senile, and the disease develops rapidly. So far, the pathogenesis of the disease is unknown, and there is no effective treatment. With the aging of the global population, the incidence of AD is increasing year by year, which has become a major disease threatening the health of the elderly, causing a serious burden to families and society, so the treatment of AD has been an urgent problem to be solved. Chromatin regulators (CRs) are an indispensable regulatory element in epigenetics. According to their role in epigenetics, CRs can be divided into three categories: DNA methylating agents, histone modifiers, and chromatin remodeling factors. But these three categories are closely related to each other when it comes to biological processes. Further studies have shown that the abnormal expression of CRs is related to a variety of biological processes, including inflammation, apoptosis, autophagy, and proliferation, indicating that the imbalance of CRs may lead to the development of many diseases, including cancer (Lu et al. 2018; Smits et al. 2020; You et al. 2015). However, at present, there are few studies on CRs, especially in non-tumor diseases. Our study is to identify CRs associated with AD.

## Materials and Methods

### Data Download and Screening of Differential CRs

We downloaded the related data sets GSE97760 (Naughton et al. 2014) and GSE138260 (Nitsche et al. 2021) (Gene Expression, Omnibus, GEO, https://www.ncbi.nlm.nih.gov/geo/) of AD patients from the GEO database, and used the sva (Leek et al. 2012) package in R software to eliminate the batch effect of the two data sets to standardize the gene expression profile. We obtained CRs from previous studies, obtained the expression data of related CRs, and used the limma (Ritchie et al. 2015) package in R software to obtain differentially expressed CRs according to the standards of *P* value < 0.05. Volcanic map and expression heatmap were used to visualize the expression of differential CRs among different samples.

### Functional Enrichment Analysis and PPI Analysis of Differentially Expressed CRs

We used R software to analyze the functional enrichment of the differential CRs files obtained in the previous step. We used org.Hs.eg.db package, clusterProfiler package, enrichplot package, and DOSE package for Gene Ontology (GO) analysis, Kyoto Encyclopedia of Genes and Genomes (KEGG) analysis, and Disease Ontology (DO) analysis, and used ggplot2 package to visualize the results. Adjusted *P* value < 0.05 was considered to be significant enrichment. We submit the differential CRs to the string database (http://www.string-db.org/) to get detailed information about gene interactions. Cytoscape software was used to draw the gene network map, and cytoHubba plug-in was used to calculate node scores, with the top 10 genes as hub genes.

### Using Single Sample Gene Set Enrichment Analysis (ssGSEA) Algorithm to Analysis Immune Correlation

In order to understand the difference of immunity between AD samples and normal samples, we used ssGSEA algorithm to calculate the expression of immune cells and immune function in related samples, and represented by correlation heat map. We analyzed the correlation and difference between immune cells and immune function in order to explore the changes of immune cells and immune function in AD.

### Analysis of the Correlation Between Hub Gene and Immune Cells and Function and the Construction of Disease Prediction Model

In order to understand the correlation between hub gene and immunity, we screened the hub gene and obtained the hub gene related to immunity. Then, we constructed a nomogram to predict the effect of the expression of related genes on the probability of disease occurrence, and used the calibration curve to evaluate the accuracy of the model.

### Prediction of Related Drugs and miRNAs in Enrichr Database

We uploaded five selected hub genes related to immunity to Enrichr database (https://maayanlab.cloud/Enrichr/), and used DSigDB database and TargetScan database to predict related drugs and miRNAs.

## Results

### Identification of Differentially Expressed CRs

**Fig. 1:**
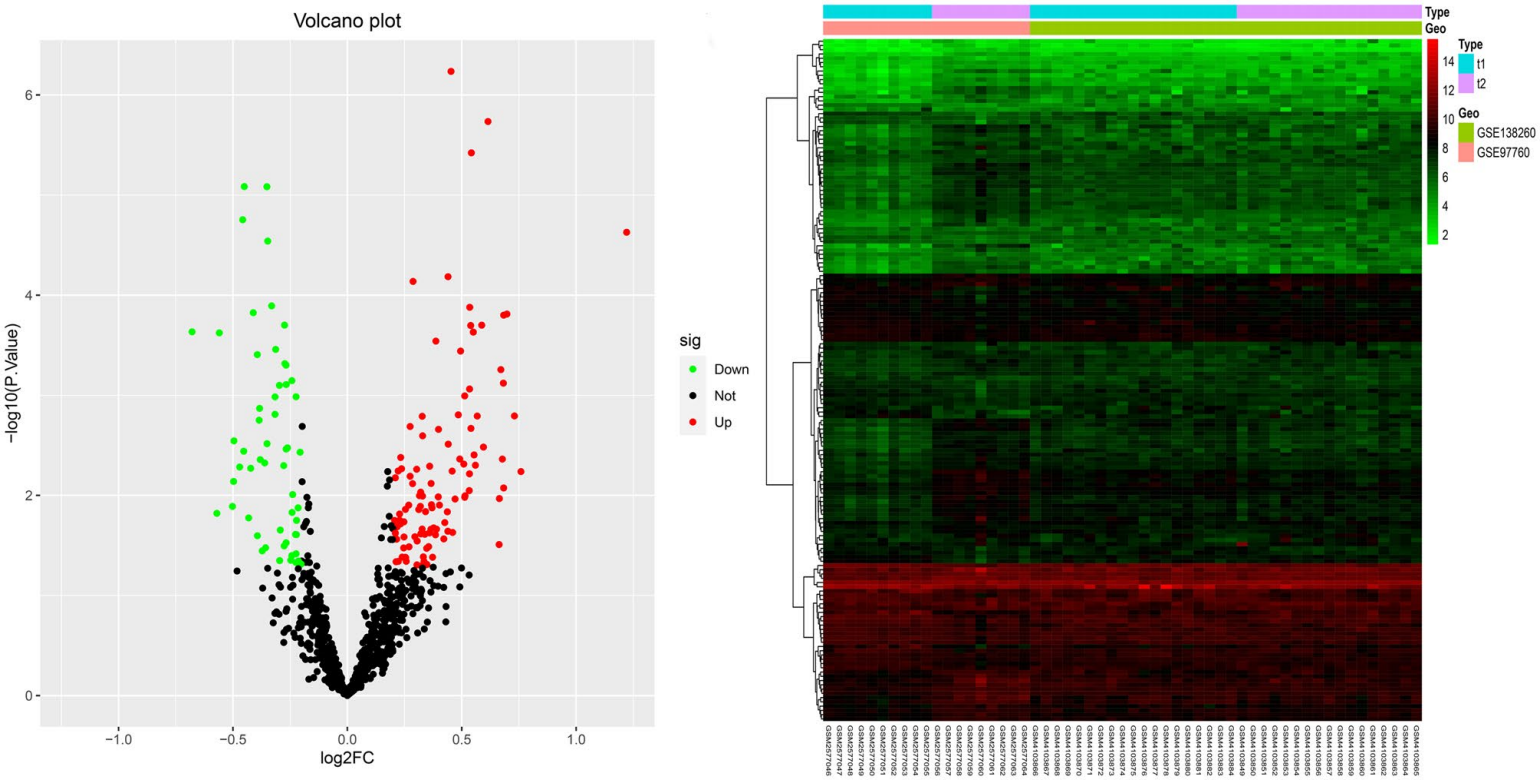
Heatmap and volcanic map showed differentially expressed CRs

We obtained 26 AD samples and 29 normal samples from the two data sets, and screened 160 differentially expressed CRs, including 115 upregulated genes and 68 downregulated genes (Fig. 1).

### Functional Enrichment Analysis and PPI Analysis of Differential CRs

**Fig. 2:**
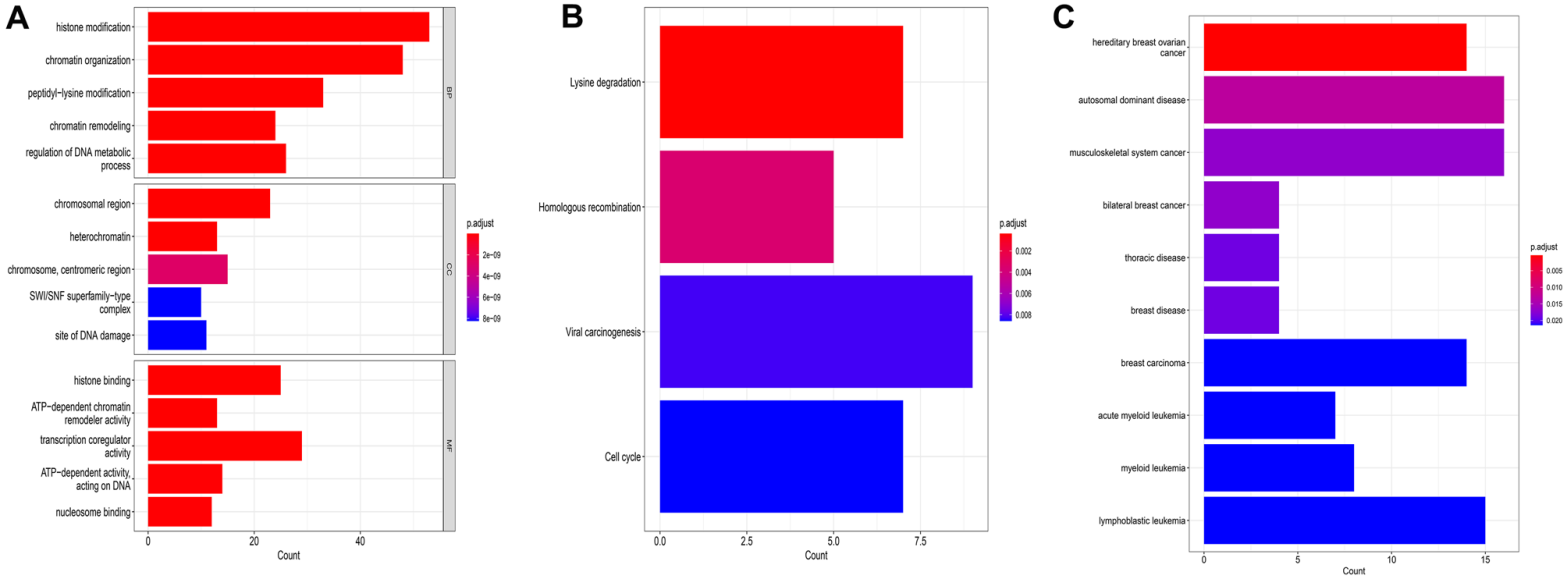
Enrichment analyses of differentially expressed CRs. **A** GO analysis; **B** KEGG analysis; **C** DO analysis

**Fig. 3:**
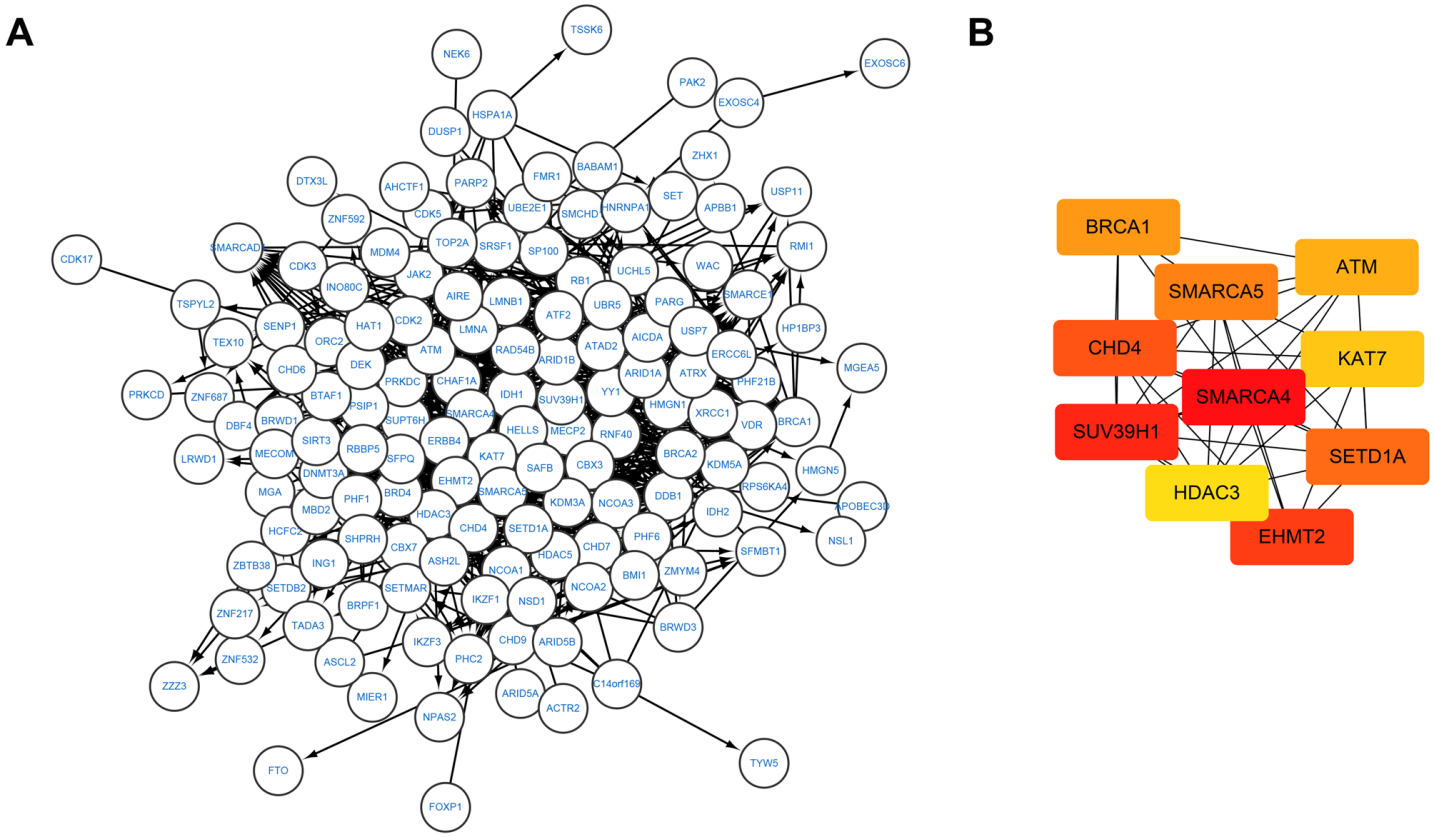
**A** PPI network of differentially expressed CRs; **B** the Hub genes

In order to understand the detailed functional information of differential CRs, we carried out GO analysis, KEGG analysis, and DO analysis. We can see the enrichment of genes in the corresponding biological processes, related pathways, and related diseases (Fig. 2). In GO analysis, we can see that CRs is mainly related to biological processes (BP) and molecular functions (MF), such as histone modification, chromatin organization, peptidyl-lysine modification, and transcription coregulator activity. In KEGG analysis, we found that differential CRs is mainly involved in viral carcinogenesis signal pathway, lysine degradation signal pathway, cell cycle, and homologous recombination. In DO analysis, differential CRs was related to autosomal dominant disease, musculoskeletal system cancer, lymphoblastic leukemia, breast carcinoma, and hereditary breast ovarian cancer. We analyzed the differential genes by PPI, and screened out the top 10 hub motifs using the cytoHubba plug-in in cystoscope (Fig. 3 and Table 1).

**Table 1:** The top 10 hub genes

Node name	MCC
SMARCA4	67,868
SUV39H1	51,103
EHMT2	45,712
CHD4	37,379
SETD1A	36,938
SMARCA5	36,914
BRCA1	20,418
ATM	19,623
KAT7	16,660
HDAC3	15,678

### Correlation Analysis Between Immune Cells and Immune Function

**Fig. 4:**
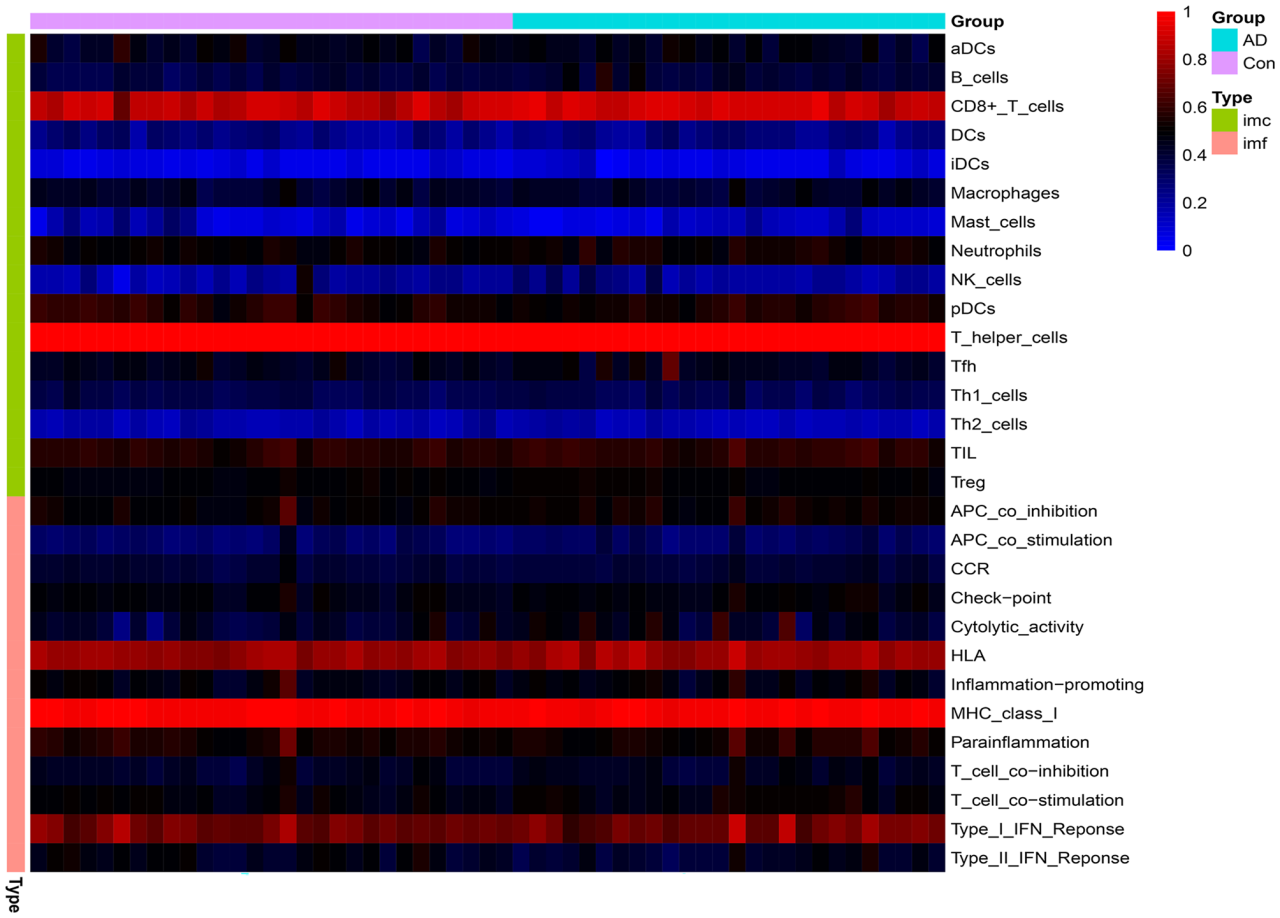
Heatmap showed the expression of immune cells and immune functions

**Fig. 5:**
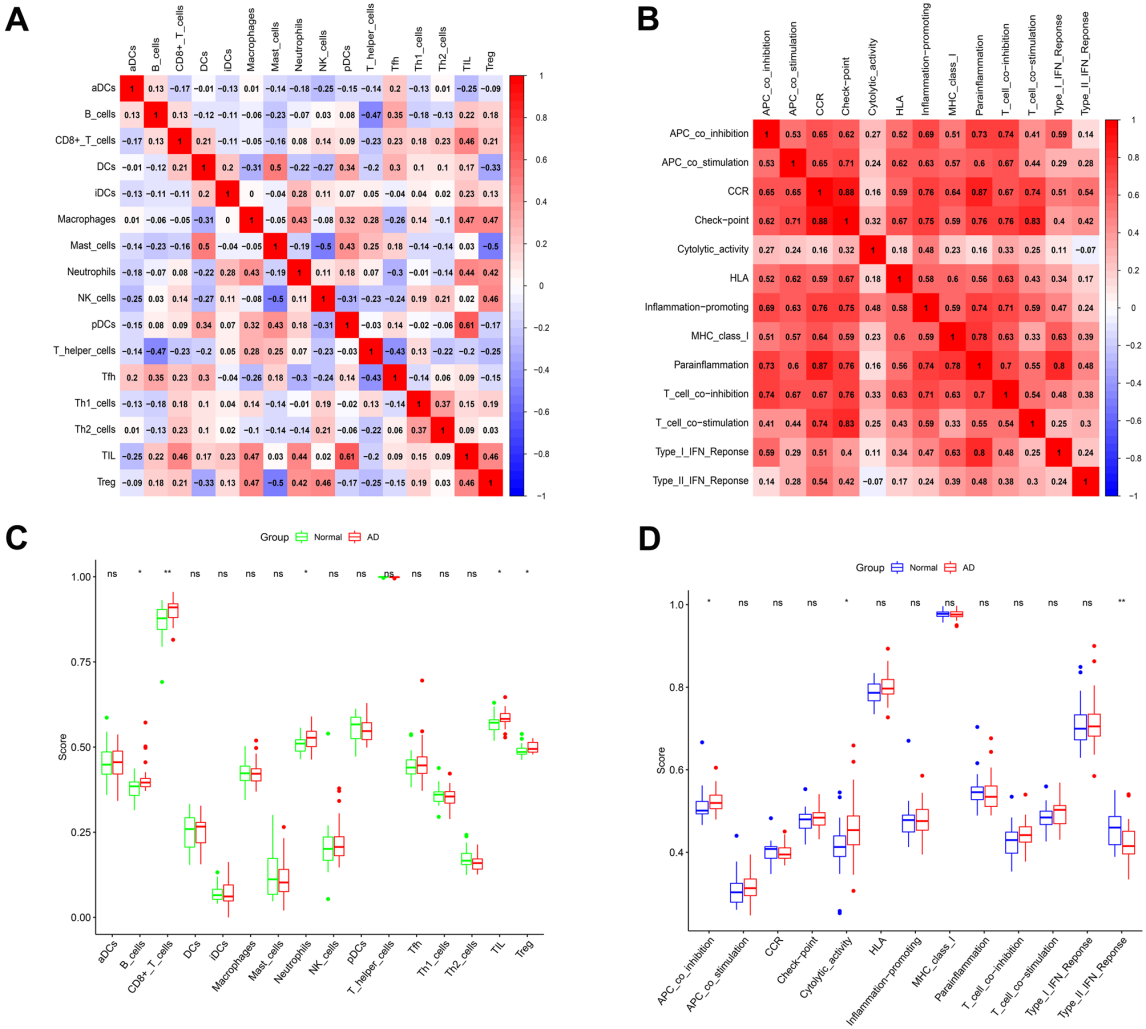
Analysis of immune cells and immune function. **A**–**B** Correlation analysis of immune cells and immune function; **C**–**D** differences in immune cells and immune function between AD samples and normal samples

We used ssGSEA algorithm to get the expression of immune cells and immune function in the sample (Fig. 4), and then we analyzed the correlation and difference between immune cells and immune function (Fig. 5). In immune cells, the correlation between TIL and pDCs reached 0.61 and the correlation between mast cells and DCs reached 0.50, suggesting that there was a strong positive correlation between them. The correlation between NK cells and Mast cells was − 0.50. The correlation between bamboo cells and T helper cells was − 0.47, suggesting that they had a strong negative correlation. In the correlation analysis of immune function, we can see that except for the weak negative correlation between type II IFN response and cytolytic activity, there is a positive correlation between immune function. Among them, between checkpoint and CCR, between CCR and para-inflammation, between T cell co-stimulation and checkpoint, there is a strong positive correlation between type I IFN response and para-inflammation, reaching 0.88, 0.87, 0.83, and 0.80, respectively. There were also differences in the expression of different immune cells and immune functions between normal samples and patients with AD. It can be seen that there are significant differences in B cells, CD8 + T cells, neutrophils, TIL, and Treg in immune cells, and T APC co-inhibition, cytolytic activity, and type II IFN response in immune function.

### The Construction of Disease Prediction Model

**Fig. 6:**
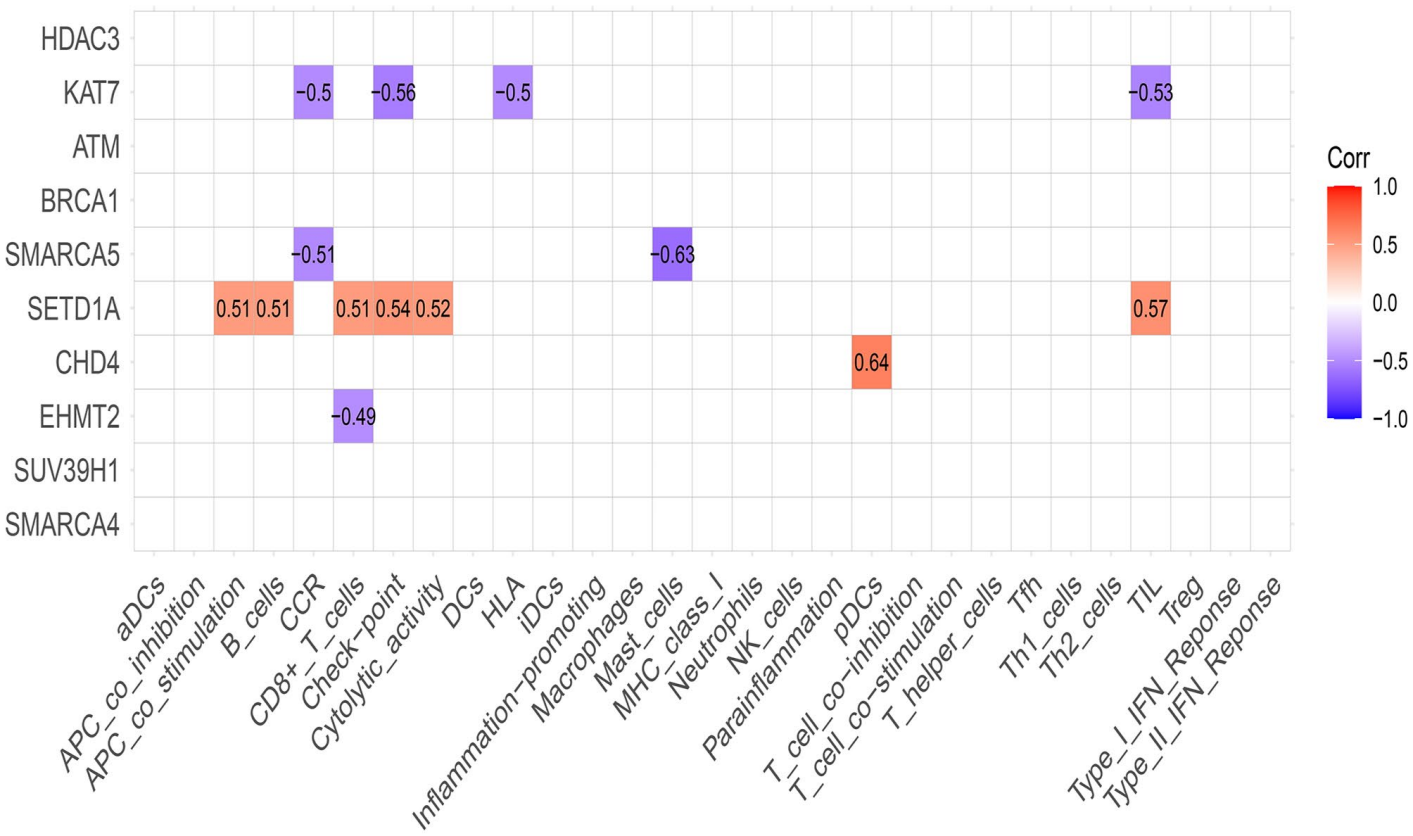
Correlation analysis of hub genes with immune cells and immune function

**Fig. 7:**
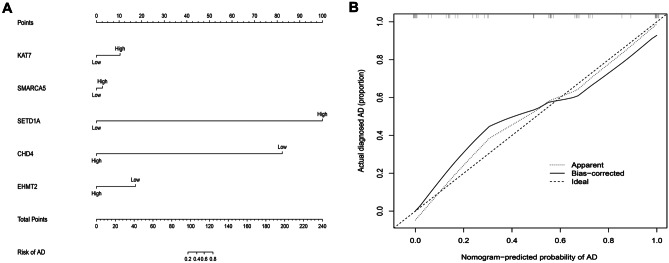
Construction of a nomogram. **A** Nomogram for predicting the risk of AD; **B** the calibration plots for predicting the risk of AD

We conducted correlation analysis on the 10 selected hub genes with immune cells and immune function. With correlation absolute value greater than 0.4 as the standard, 5 immune-related genes were finally identified, among which KAT7 was negatively correlated with immune cell TIL and immune function CCR, checkpoint, and HLA. SMARCA5 was negatively correlated with mast cells and CCR. SETD1A was positively correlated with immune cells B cells, CD8 + T cells, TIL, APC co -stimulation, checkpoint, and cytolytic activity. There was a positive correlation between CHD4 and immune cell pDCs. EHMT2 was negatively correlated with CD8 + T cells. This suggests that relevant chromatin regulators may be involved in the immune response of AD (Fig. 6). We used nomograph to construct a disease prediction model, which showed that the higher the expression of KAT7, SMARCA5, and SETD1A, the higher the probability of developing AD, and the higher the expression of CHD4 and EHMT2, the lower the probability of developing AD (Fig. 7). The calibration curve also indicates that the model has a good predictive function.

### Prediction of Related Drugs and miRNAs

**Fig. 8:**
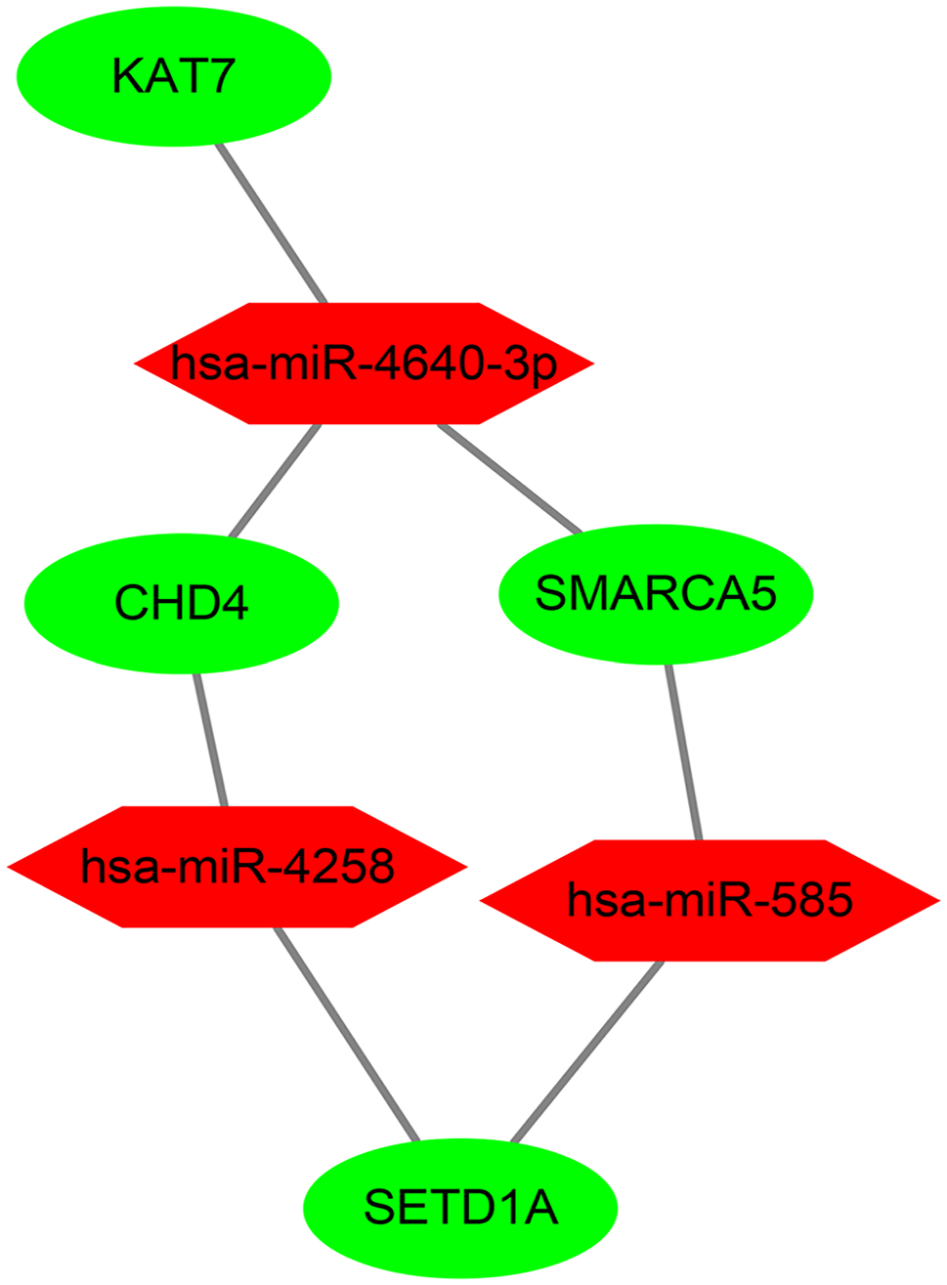
The network diagram of related miRNAs

We upload genes to Enrichr database, use DSigDB database to predict related drugs, and use TargetScan database to predict related miRNA. According to *P* value < 0.05 as the standard, we have obtained 520 kinds of related drug (Table 2), and 3 kinds of related miRNA (Table 3) and network diagram (Fig. 8), which may be useful for finding therapeutic targets for AD.

**Table 2:** Drugs associated with selected genes (top 10 drugs)

Term	*P*-value	Genes
deferoxamine CTD 00,005,759	3.71E-04	EHMT2; SETD1A
brimonidine CTD 00,000,810	0.002747205	EHMT2
MALEIMIDE CTD 00,001,979	0.002747205	EHMT2
quinolinic acid CTD 00,007,181	0.002747205	EHMT2
Pyrrolidine dithiocarbamate TTD 00,010,430	0.002747205	EHMT2
Quisqualate TTD 00,010,472	0.002747205	EHMT2
2'-Hydroxychalcone TTD 00,000,428	0.002747205	EHMT2
buspirone CTD 00,005,542	0.002747205	EHMT2
Carbidopa hydrate TTD 00,002,683	0.002747205	EHMT2
ergocalciferol TTD 00,007,877	0.002747205	EHMT2

**Table 3:** miRNAs associated with selected genes

Term	*P*-value	Genes
hsa-miR-4640-3p	0.00642384	SMARCA5; KAT7; CHD4
hsa-miR-585	0.00999761	SETD1A; SMARCA5
hsa-miR-4258	0.032452473	SETD1A; CHD4

## Discussion

AD is a common neurological disease in the elderly, which is common in women. With the progress of the disease, patients can gradually develop cognitive impairment, as well as behavioral and personality abnormalities, resulting in the emergence of mental symptoms and gradual loss of self-care ability (Scheltens et al. 2021). The death of patients is often caused by many complications. AD is the result of gene, environment, lifestyle, and other factors. The typical histopathological changes of AD are amyloid deposition and neurofibrillary tangles in the brain. At present, there are many theories trying to explain this change, including β-amyloid, Aβ (β-amyloid β) waterfall theory, tau protein theory, and neurovascular hypothesis. But the exact etiology of AD has not been clarified. Studies have shown that the increase of follicle-stimulating hormone (follicle-stimulating hormone, FSH) may be an important reason why women are more likely to develop AD than men (Xiong et al. 2022). The treatment of AD includes drug therapy (Kabir et al. 2020), including acetylcholinesterase inhibitors to improve cognitive function, N-methyl-D-aspartate receptor antagonists, brain metabolic activators, antipsychotics, and antidepressants to control mental symptoms. Others include exercise therapy (Valenzuela et al. 2020), music therapy (Leggieri et al. 2019), and so on. With the continuous development of technology, immunotherapy for AD is a treatment with great potential for development and many patients benefit from this (Adolfsson et al. 2012; Bohrmann et al. 2012; Sevigny et al. 2016).

CRs are a class of enzymes with special functional domains, which can recognize, form, and maintain epigenetic state in an environment-dependent way. Chromatin regulatory factors can dynamically regulate chromatin structure in response to internal and external signals. Therefore, chromatin regulatory factor is expected to become a new target for the treatment of many diseases ^13,14^. Studies have shown that chromatin regulatory factors are associated with a variety of cancers (Shu et al. 2012; Zhou et al. 2010; Zhu et al. 2022), but there are few studies in other diseases. In this study, we first obtained the relevant data sets of patients with AD from the GEO database and screened 160 differentially expressed CRs between AD samples and normal samples. We systematically analyzed the biological pathways of CRS and constructed a PPI network, and screened 10 hub genes according to node scores. Then, we combine the hub gene with immune binding analysis to get the genes related to immunity. We have established a risk model related to the disease probability, and the calibration curve shows that the model has good prediction ability. Finally, we uploaded the related genes to the database to get the related drugs and miRNAs. GO analysis showed that the difference CRS was mainly related to histone modification, chromatin organization, peptidyl-lysine modification, transcription coregulator activity, and so on. The results of KEGG analysis emphasized the differential CRS in homologous recombination, cell cycle, lysine degradation signal pathway, and viral carcinogenesis signal pathway. This indicates that differential CRS is involved in a variety of biological processes. In DO analysis, the enrichment of differential CRs in autosomal dominant disease was the most significant, and there was a strong correlation. Familial AD is a euchromatin-dominant hereditary disease, which suggests that differential CRs may be involved in the pathogenesis of AD. In addition, our screening of hub genes related to immunization also suggests that CRs may be related to the immune process of AD.

KAT7 gene is a newly discovered gene, which was initially found that deletion can delay aging (Wang et al. 2021b). Existing studies have shown that the lack of KAT7 can lead to abnormal brain development^19^, and it has also been confirmed to be associated with gastric cancer (Jie et al. 2020), colon cancer (Taniue et al. 2020), non-small cell lung cancer (Gao et al. 2021), rheumatoid arthritis (Gao et al. 2017), and other diseases. SMARCA5 is a circular RNA that has been shown to be associated with a variety of cancers (Miao et al. 2020; Tan et al. 2019; Tong 2020; Zhang et al. 2021); previous studies have shown that there is no significant difference in SMARCA5 methylation frequency between the young, the elderly, and the AD group. This indicates that SMARCA5 may have nothing to do with AD (Silva et al. 2008). Some studies have also shown that mutations in SMARCA5 can lead to a series of delayed neural development, which provides a new biomarker for genetic diagnosis (Li et al. 2021). In our study, immune correlation analysis showed that there was no significant difference in immune cells and immune function related to SMARCA5 between AD samples and normal samples. SETD1A, a lysine-methyltransferase (lysine-methyltransferase), has been shown to be a risk gene for schizophrenia associated with cognitive impairment, and its mutation increases the risk of schizophrenia (Mukai et al. 2019; Singh et al. 2016). Enhanced SETD1A is associated with the occurrence and metastasis of a variety of tumors (Kang et al. 2021; Wu et al. 2020; Yang et al. 2020). SETD1A can also prevent aging by regulating mitotic gene expression procedures (Tajima et al. 2019). CHD4 is an important component of Mi-2/nucleosome remodeling and deacetylase complex, which participates in DNA repair after injury. Recent studies have shown that CHD4 has carcinogenic function, suppresses a variety of tumor suppressor genes through epigenetic regulation, and participates in the development of a variety of tumors (Chang et al. 2021; Li et al. 2018; Xia et al. 2017). It has been proved that it can control gene expression in embryonic stem (ES) cells (Lai and Wade 2011). CHD4 is also related to cardiac development (Sifrim et al. 2016) and neurodevelopmental disorders (Trinh et al. 2019). In our study, we also found no correlation between CHD4 and the immune process of AD. EHMT2 is a key epigenetic regulator of neuronal function. Studies have shown that the cognitive decline of AD is caused by the loss of glutamate receptors. EHMT2 inhibitors can reduce the content of H3K9me2, thus saving the synaptic and cognitive function of AD (Zheng et al. 2019). EHMT inhibition can also reduce the level of hyperphosphorylated tau, thus improving AD symptoms (Wang et al. 2021a), but the relationship between EMHT2 and AD immunity has not been studied in detail. Our prediction model preliminarily revealed the five related CRs of KAT7, SMARCA5, SETD1A, CHD4, and EHMT2, and also obtained possible drugs and related miRNAs, which provided some ideas for the treatment of AD.

Of course, there are some deficiencies in our research, and we need to explore the specific mechanism through further experiments. In addition, the nomogram shows that the lower the expression of EHMT2 gene, the higher the risk of AD, which seems to be in conflict with previous studies, which may be related to our sample error. Suppression of the EHMT2 gene has been shown to improve symptoms in AD mice, but the correlation between EHMT2 and the risk of AD may need to be verified by further experiments.

## Conclusion

In conclusion, we have identified five CRS that may be related to the immunity of AD patients: KAT7, SMARCA5, SETD1A, CHD4, and EHMT2. Our study needs to be verified by further experiments.

## Data Availability

The original contributions presented in the study are included in the article/supplementary material. Further inquiries can be directed to the corresponding authors.
